# Intent disclosure in late-life suicide: Age group differences in correlates and associations with suicide means

**DOI:** 10.3389/fpsyg.2022.949333

**Published:** 2022-10-05

**Authors:** Namkee G. Choi, C. Nathan Marti

**Affiliations:** Steve Hicks School of Social Work, University of Texas at Austin, Austin, TX, United States

**Keywords:** late-life suicide, suicide means, suicide prevention, intent disclosure, physical and mental health, relationship/other life stressors

## Abstract

Age-adjusted suicide rates declined from 2018 to 2020. However, suicide rates among older adults, particularly males 75 and older, have continued to rise, and the evidence base for effective interventions to prevent suicide in late life remains limited. One strategy to prevent older adults’ suicidal behavior is to intervene when they reveal suicide intent. Previous research found that a significant proportion of older suicide decedents disclosed their suicide intent close to the fatal incident. In this study, based on the 2017–2019 United States National Violent Death Reporting System (NVDRS) data, we examined: (1) correlates of intent disclosure among three age groups (65–74, 75–84, and 85+) of older suicide decedents (*N* = 17,917; 14,856 men and 3,061 women); and (2) associations of suicide means with intent disclosure and suicide contributing factors. The results show that 19.9% of all suicide decedents aged 65+ (18.7%, 21.0%, and 22.0% in the 65–74, 75–84, and 85+ age groups, respectively) disclosed their suicide intent to their partner, family/friends, and healthcare providers within a month of their death. Multivariable analyses using generalized linear models for a Poisson distribution with a log link showed that physical and mental health, substance misuse, addiction problems, and relationship/other life stressors were associated with a higher likelihood of intent disclosure in the 65–74 and 75–84 age groups. However, only physical health problems were associated with a higher likelihood of intent disclosure among those aged 85 and older. Intent disclosure was not associated with using firearms and poisoning as suicide means but with a lower likelihood of hanging or suffocation. Mental health and substance misuse problems were associated with higher odds of hanging or suffocation and poisoning, and physical health problems and male sex in the 85+ age group were associated with higher odds of firearm use. Suicide prevention strategies for those who have disclosed their suicide intent or are at risk of suicidal behavior should include more patient-centered comfort and palliative care, mental health/substance misuse/addiction treatment, and restriction of access to potential suicide means. More research on older adults who disclose suicide intent and late-life suicide prevention strategies is needed.

## Introduction

Recent suicide mortality data from the United States Centers for Disease Control and Prevention (CDC) shows that after increasing from 2000 through 2018, age-adjusted suicide rates declined from 2018 (14.2 per 100,000) to 2020 (13.5 per 100,000; Garnett et al., 2022). Despite the overall decreasing trend, rates among men 75 years and older continued to increase and have been the highest of all age groups (e.g., 40.1 per 100,000 in 2020) over the past two decades (Garnett et al., 2022). High late-life suicide rates are attributed to a greater intent to die, greater premeditation, use of more deadly means and giving fewer warnings to others of their suicidal intentions among older adults compared to younger age groups (Conwell et al., 1998; Conner et al., 2019).

Systematic reviews identify risk factors for completed late-life suicide to include male sex, violent methods of self-harm (e.g., firearms, hanging/suffocation), any psychiatric disorders (e.g., depression, anxiety, and bipolar disorders), physical health problems (e.g., cancer, chronic diseases, chronic pain), neurological disorders, functional disability, stressors/bereavement, living alone, and limited social connectedness (Fässberg et al., 2012, 2016; Beghi et al., 2021). Systematic and narrative reviews also identified psychiatric comorbidity (e.g., depression and anxiety, mental disorders with substance use problems), psychiatric and physical multimorbidity, cognitive impairment, neurocognitive disorders, reduced social support, and loneliness as contributors to suicidal ideation and behaviors in late life (Conejero et al., 2018; Xiong et al., 2020; Fernandez-Rodrigues et al., 2022). A recent study of Medicare fee-for-service beneficiaries 65 and older also found significantly increased suicide rates following a diagnosis of Alzheimer’s and related dementia, especially among those age 65–74 years and during the first 90 days after diagnosis (Schmutte et al., 2022). Other studies showed that among depressed older adults, suicidal individuals performed significantly worse than non-suicidal individuals on the cognitive tests, and deficits in executive functioning and other cognitive domains predicted serious suicidal behavior, suggesting that in some cases, late-life suicidal behavior is possibly reflective of a dementia prodrome (Szanto et al., 2020; Gujral et al., 2021; Richard-Devantoy et al., 2021).

Given high rates of late-life suicide, early detection of suicidal ideation and provision of mental health treatment and other support for older adults who suffer from psychiatric, physical, and social health problems is essential for preventing late-life suicide. One way to reduce premature mortality from suicides is also to intervene and manage risks when individuals in distress disclose suicidal intent. Studies have shown that one reason for such disclosure, to mostly one’s confidants and others in the informal social support network, is help-seeking (Fulginiti et al., 2016; Fulginiti and Frey, 2019). However, severe suicide attempters [i.e., those whose injuries necessitated intensive care unit (ICU) admission], compared to suicide ideators and mild suicide attempters (i.e., those who did not require ICU admission), tend to lack willingness to self-disclose (Apter et al., 2001).

A majority of older adults intending to die by suicide may deny suicidal ideation and do not disclose their intent, especially to healthcare providers (Smith et al., 2013; Husky et al., 2016), but some may still want to inform their informal support systems about their intension for various reasons. In-depth interviews with Dutch older adults deliberating on ways to end their lives at a self-chosen moment (i.e., rational suicide, self-chosen death) revealed their struggle with ambiguities and ambivalences between intending and actually performing self-directed death (or not; van Wijngaarden et al., 2016). Even after they made a putatively rational decision for suicide as a way to spare them further suffering from health problems, a majority had certain attachments to life, concerns about the emotions of family members, a dilemma associated with their spiritual beliefs, and worry and fear about the dying process (van Wijngaarden et al., 2016).

To our knowledge, little research has been done on the characteristics of older adults who disclosed their suicide intent. A study of suicide decedents 50 years and older in the 2005–2014 United States National Violent Death Reporting System (NVDRS) showed that nearly a quarter of them disclosed their suicidal intent, mostly to an intimate partner or other family members, within the last month of their life and that both depressed mood and physical health problems were associated with increased disclosure odds (Choi et al., 2017). The study also found that controlling for depressed mood, physical health problems, and other stressors that contributed to suicide, individuals age 80 and older had higher odds of intent disclosure than those age 50–59. With unabated rates of suicide among those age 75 and older, more research is needed to examine potential age group differences in intent disclosure rates and correlates of intent disclosure among older adults who died by suicide.

In this study based on the 2017–2019 NVDRS, we first examined mental health, substance misuse/addiction, physical health, and other suicide precipitants as correlates of suicide intent disclosures in three age groups (65–74, 75–84, and 85+) of older suicide decedents. We then examined associations of suicide means with intent disclosure and suicide contributing factors. We posited a hypothesis that mental health/substance misuse/addiction problems, relationship problems, and other life stressors would be significant correlates in the 65–74 age group, while physical health problems would be significant correlates in the 75–84 and 85+ age groups. Previous studies of older suicide decedents showed that significantly higher proportions of those age 65–75 than those age 75+ had mental health/substance use problems, while significantly higher proportions of those age 75+ than those age 65–74 had physical health problems as suicide precipitants (Choi et al., 2019; Schmutte and Wilkinson, 2020). These health problems as suicide precipitants may also be significant correlates of intent disclosure.

We also hypothesized that intent disclosure would be associated with suicide means; however, given the lack of previous research on the relationships among suicide means, intent disclosure, and the circumstances of death, we did not posit any directional hypothesis. Most previous research on suicide attempters found no association between suicide intent and choice of suicide means (Peterson et al., 1985; Plutchik et al., 1989), as other factors such as the availability and acceptability of methods and attempters’ knowledge of the likely lethality of a given method also play a role (Harvard T.H. Chan School of Public Health, n.d.). However, other studies found that higher levels of intent were associated with use of more lethal means (Hamdi et al., 1991; Townsend et al., 2001; Brown et al., 2004). Older adults with a high degree of suicide intent may decide to choose more lethal means; however, as discussed, intent disclosure may not be a good indicator of the degree of intent. Better understanding of health and other factors associated with intent disclosure and potential associations between intent disclosure and suicide means is needed for more effective suicide prevention. The findings will provide insights into the demographic and clinical characteristics of older-adult suicide decedents who disclosed their suicide intent and associations of suicide means with intent disclosure and suicide contributing factors in late life.

## Materials and methods

### Data source

We focused on older-adult suicide decedents in the 2017–2019 NVDRS [*N* = 17,917, ages 65–105 at the time of death; 14,856 men (82.9%) and 3,061 women (17.1%)]. NVDRS is the only state-based violent death reporting system in the United States that provides information and context on when, where, and how violent deaths occur and who is affected (National Center for Injury Prevention and Control, 2021). NVDRS links data from death certificates and reports from coroners/medical examiners (CME) and law enforcement (LE) agencies on cases of violent deaths--suicides, homicides, deaths from legal intervention (i.e., victim killed by LE acting in the line of duty), deaths of undetermined intent, and unintentional firearm deaths. CME/LE reports are from the injury/death scene, ongoing investigations, or family/friend accounts and often serve as the basis of the circumstances of death and the NVDRS variables that were “calculated” (coded “Yes” when endorsed by the CME and/or LE reports vs. “No/not available/unknown”). When available, crime lab and toxicology reports included in CME reports are also abstracted and entered in NVDRS. During the 3-year study period, 43 states, the District of Columbia, and Puerto Rico participated in NVDRS; however, not all states provided complete data for all 3 years (see NCIPC (2021) for a detailed list of participating states in each year). The authors of this study were granted access to de-identified NVDRS data for this study by the CDC’s NVDRS-Restricted Access Data (RAD) review committee. This study based on deceased individuals was exempt from the authors’ institutional review board’s review.

### Measures

#### Intent disclosure

In NVDRS, disclosure was defined as either (1) disclosure of suicidal thoughts or intent to die by suicide to another person *via* verbal, written, or electronic communications within a month (or recently) before suicide, whether explicitly (e.g., “I plan to go to my cabin with my gun and never come back”) or indirectly (e.g., “I know how to put a permanent end to this pain”); or (2) a separate suicide attempt within a month of the suicide. If the decedent disclosed intent to die by suicide only at the moment of the suicide (i.e., when there was no opportunity to intervene to stop the suicide), the NVDRS classifies this as a suicide note rather than a disclosure (CDC, 2021). Nondisclosure was defined as absence of disclosure or unknown disclosure status. NVDRS also includes data on the persons to whom decedents disclosed.

#### Suicide means

These were identified from the International Classification of Diseases 10th Revision (ICD-10) codes for intentional self-harm (X60-X84) for underlying cause of death in death certificates and/or from the underlying cause descriptions in CME reports. They included the following: firearms; hanging/suffocation; poisoning due to any type of alcohol/drug/medicine/chemical overdose or with gas (e.g., carbon monoxide, nitrogen); laceration/sharp instruments; blunt objects; jumping from heights; contact with moving objects (train/other vehicles); drowning; and other (fire, hypothermia, electrocution, starvation, dehydration, not adhering to or refusing medical care, or undetermined causes). We classified them into four categories in this study: firearms, hanging/suffocation, poisoning, and other.

#### History of suicide attempts

This referred to any previous suicide attempt before the fatal incident (i.e., including any in the past month), regardless of the severity and injury status.

***Mental health and substance misuse/addiction*** (without the need for any indication that they directly contributed to the death): Mental health problems included: (1) depressed mood at the time of death (without the need for a clinical diagnosis); and (2) any diagnosed mental health problem [disorders and syndromes listed in DSM-5 (American Psychiatric Association, 2013)] at the time of death. Substance misuse/addiction problems included: (1) alcohol problem/addiction; and (2) other substance misuse/addiction (e.g., prescription drug misuse, chronic/abusive/problematic marijuana use, any use of other illicit drugs or inhalants). Additionally, we included any other addiction (e.g., gambling, sex) that appears to have contributed to the death. We also reported any history of mental health/substance use treatment for descriptive purposes only [as the data on treatment status are likely to be incomplete as they were reported by family/friends/other informants, not from healthcare professionals or official medical records (email communication with the NVDRS-RAD team; April 19, 2022)].

#### Contributing physical health problem

In NVDRS, this was recorded “Yes” if any diagnosed or perceived physical health problem (e.g., terminal disease, debilitating condition, chronic pain) was relevant to the death (e.g., “despondent over recent diagnosis of cancer” or “complained that he could not live with the pain associated with a condition” even if the condition may not have been diagnosed or existed).

#### Contributing relationship and other life stressors

These included: (1) relationship problems (conflict with an intimate partner and/or other family members, arguments, other family stressors, caregiver burden, or abuse by a caregiver); (2) recent suicides or other deaths of family/friends or traumatic anniversary; (3) job/finance/housing problems; and (4) criminal/civil legal problems.

#### Number of crises

NVDRS provides a variable that is the count of crises (“current/acute event within two weeks of death”) that the decedent faced with respect to mental health, substance misuse/addiction, physical health, and relationship and other life stressors discussed above.

#### Demographic variables

Data on age at the time of death, sex, race/ethnicity, level of education, marital status, and military service status were from the death certificates and CME/LE reports. Census region of residence was examined for descriptive purposes only.

## Analysis

All statistical analyses were performed using Stata/MP 17. First, single variable multinomial logistic regression analyses were used to examine any differences between the 65–74 age group and two older age (75–84 and 85+) groups in demographic and other variables of interest. Second, to test the study hypothesis regarding correlates of suicide intent disclosure in each age group, we fit three generalized linear models (GLMs) for a Poisson distribution with a log link. We fit GLMs rather than logistic regression models because odds ratios exaggerate the true relative risk to some degree when the event (i.e., intent disclosure in this study) is a common (i.e., >10%) occurrence (Grimes and Schulz, 2008). The independent variables for all three GLM models were mental health problems, substance misuse/addiction problems, contributing physical health problems, relationship/other life stressors, and demographics. Third, to examine associations between intent disclosure and suicide means, we also fit three GLMs for firearm use, hanging/suffocation, and poisoning as the dependent variables. As a preliminary diagnostic, we used variance inflation factor (VIF), using a cut-off of 2.50 (Allison, 2012), from linear regression models to assess multicollinearity among covariates. VIF diagnostics indicated that multicollinearity was not a concern. GLM results are reported as incidence rate ratios (IRRs) with 95% confidence intervals (CIs). Significance was set at *p* < 0.05.

## Results

### Demographic characteristics, intent disclosure, and suicide means by age group

Table 1 shows that of the study sample, 54.7% were age 65–74, 31.2% age 75–84, and 14.1% age 85+. Compared to those age 65–74, two older groups of decedents had significantly higher percentages of men but lower percentages of Black/African Americans, and the 85-and-older group also had significantly lower percentages of Hispanics and the residents of the Southern region but a higher percentage of Asian/Pacific Islanders. Two older groups included lower percentages of divorced/separated/never married and college-educated individuals but higher percetages of widowed individuals, veterans, and those who died at home.

**Table 1:** Demographic characteristics, intent disclosure, and suicide means of suicide decedents age 65+: Results from single-variable multinomial logistic regression models.

	65–74 years *N* = 9,797 (54.7%)	75–84 years *N* = 5,592 (31.2%)	85+ years *N* = 2,528 (14.1%)	75–84 years vs. 65–74 years RRR (95% CI)	85+ years vs. 65–74 years RRR (95% CI)
Demographics					
Sex (%)					
Female	20.7	13.2	11.6	–	–
Male	79.3	86.8	88.4	1.71 (1.56–1.87)^***^	1.98 (1.74–2.26)^***^
Race/ethnicity (%)					
Non-Hispanic White	90.5	91.5	93.0	–	–
Black/African American	2.9	2.4	1.5	0.81 (0.65–0.99)^*^	0.50 (0.36–0.71)^***^
Hispanic	3.8	3.3	2.3	0.86 (0.72–1.03)	0.59 (0.45–0.79)^***^
Asian/Pacific Islander	1.9	2.2	2.7	1.12 (0.89–1.42)	1.34 (1.01–1.78)^*^
Other	0.9	0.6	0.5	0.71 (0.48–1.05)	0.61 (0.35–1.08)
Marital status (%)					
Married	44.3	47.3	34.5	–	–
Divorced/separated	30.2	21.5	11.4	0.66 (0.61–0.72)^***^	0.48 (0.42–0.56)^***^
Widowed	12.2	25.	49.9	1.91 (1.75–2.09)^***^	5.23 (4.70–5.82)^***^
Never married/single-unspecified	11.8	5.2	3.7	0.42 (0.36–0.48)^***^	0.41 (0.33–0.51)^***^
Unknown	1.5	1.0	0.5	0.63 (0.46–0.85)^**^	0.43 (0.24–0.75)^**^
Education (%)					
=<High school	47.8	56.1	58.7	–	
Some college/associate’s degree	23.0	17.6	13.9	0.65 (0.60–0.71)^***^	0.49 (0.43–0.56)^***^
Bachelor’s degree or higher	26.0	23.4	24.9	0.77 (0.71–0.83)^***^	0.78 (0.70–0.87)^***^
Unknown	3.2	2.9	2.5	0.77 (0.63–0.93)^**^	0.63 (0.47–0.83)^**^
Military service (%)					
No, not available, unknown	68.5	55.6	36.4	–	–
Yes	31.5	44.4	63.6	1.74 (1.62–1.86)^***^	3.80 (3.47–4.16)^***^
Injury location (%)					
Not at home or unknown	21.3	16.3	12.6	–	–
At home	78.7	83.7	87.4	1.39 (1.27–1.51)^***^	1.87 (1.65–2.12)^***^
Census region of residence					
Northeast	16.4	16.2	17.8	–	–
Midwest	25.7	25.6	26.4	1.01 (0.91–1.12)	0.95 (0.83–1.08)
South	27.9	27.3	23.9	0.99 (0.89–1.10)	0.79 (0.69–0.90)^**^
West	28.6	29.7	31.0	1.05 (0.95–1.16)	1.00 (0.87–1.14)
Puerto Rico/other United States territories/unknown	1.4	1.2	0.9	0.85 (0.63–1.15)	0.57 (0.36–0.90)^*^
Disclosed suicidal intent within last month (%)					
No, not available, unknown	81.3	79.0	78.0	–	–
Yes	18.7	21.0	22.0	1.16 (1.07–1.26)^***^	1.23 (1.11–1.37)^***^
Disclosed to whom (among those who disclosed; *N* = 3,564) (%)					
Previous or current intimate partner	34.9	33.9	24.6		
Other family member	32.4	41.6	44.9		
Friend/colleague	11.0	7.4	8.1		
Neighbor	3.1	3.2	4.1		
Healthcare worker	7.1	6.5	10.4		
Other disclosure *via* social media/other electronic means	0.1	0	0.2		
Other	11.8	8.2	9.5		
Unknown	5.4	4.8	5.0		
Left suicide note/electronic message (%)					
No, not available, unknown	67.1	70.7	68.8	–	–
Yes	32.9	29.3	31.2	0.84 (0.79–0.91)^***^	0.92 (0.84–1.02)
Suicide means (%)					
Firearms	62.7	74.7	74.3	–	–
Hanging, strangulation, or suffocation	14.6	9.6	9.7	0.55 (0.49–0.61)^***^	0.56 (0.48–0.65)^***^
Drug, other chemical, or gas poisoning	15.7	10.4	10.5	0.56 (0.50–0.62)^***^	0.57 (0.50–0.66)^***^
Other^1^	7.0	5.3	5.5	0.63 (0.55–0.73)^***^	0.66 (0.54–0.79)^***^

1 Jump from a high place, blunt force from moving vehicle/train/other, sharp or blunt object, drowning, smoke/fire/flame/electrocution/hypothermia, other means, or unknown.*p < 0.05; **p < 0.01; ***p < 0.001.

Table 1 also shows that 18.7%, 21.0%, and 22.0% in the 65–74, 75–84, and 85+ age groups, respectively, disclosed their suicide intent, and the average for all decedents 65 years and older was 19.9%. The difference in the percentages between those age 65–74 and two older groups was statistically significant. Two thirds to three quarters of disclosures were to a previous or current intimate partner and/or other family members. A little over 10% of the disclosers in the 85+ age group disclosed their intent to a healthcare provider. In all three age groups, a little less than a third left a suicide note, but a significantly lower proportion of the 75–84 age group the 65–74 age group did so. Additional analysis showed no significant difference between those who disclosed intent and those who did not in leaving a suicide note in all three age groups. Additional analysis also showed significant regional differences in intent disclosure [12.0%, 26.0%, 27.5%, 33.7%, 0.8% in the Northeast, Midwest, South, West, and Puerto Rico/other territories/unknown, respectively, Pearson *χ*^2^(df = 4) = 96.09, *p* < 0.001].

With respect to suicide means, 62.7%, 74.7%, and 74.3% in the 65–74, 75–84, and 85+ age groups, respectively, used firearms. The rates were 14.6%, 9.6%, and 9.7% for hanging/suffocation; 15.7%, 10.4%, and 10.5% for poisoning; and 7.0%, 5.3%, and 5.5% for other means. However, Table 2 shows that sex differences in suicide means were significant in all three age groups. More women used poisoning (41.0%, 39.6%, and 43.2% in the 65–74, 75–84, and 85+ age groups, respectively) than firearms, whereas most men (70.2%, 88.8%, and 80.3% in the 65–74, 75–84, and 85+ age groups, respectively) used firearms. Additional analyses showed no significant difference in suicide means by age group among women [Pearson *χ*^2^(df = 6) = 6.26, *p* = 0.392], but significant difference by age group among men [Pearson *χ*^2^(df = 6) = 224.35, *p* < 0.001].

**Table 2:** Suicide means by age group and sex (%).

	All (*N* = 17,917)		65–74 years (*N* = 9,797)		75–84 years (*N* = 5,592)		85+ years (*N* = 2,528)	
	Male *n* = 14,856	Female *n* = 3,061	Male *n* = 7,770	Female *n* = 2,027	Male *n* = 4,852	Female *n* = 750	Male *n* = 2,234	Female *n* = 294
Firearm	75.2	33.7	70.2	33.9	88.8	35.0	80.3	28.2
Hanging/suffocation	11.6	15.9	14.4	15.5	8.6	15.7	8.4	19.4
Poisoning	7.6	40.8	9.0	41.0	5.9	39.6	6.3	43.2
Other^1^	5.6	9.6	6.4	9.6	4.7	9.7	5.0	9.2
Sex difference: *p*-value (Pearson *χ*^2^)	*p* < 0.001		*p* < 0.001		*p* < 0.001		*p* < 0.001	

1 Including sharp or blunt object (2.2% of all decedents); jump from a high place (1.7%); drowning (0.9%); blunt force from moving vehicle/train/other (0.8%); smoke/fire/flame/electrocution/hypothermia (0.4%); medical device discontinuation (0.03%); intentional starvation/dehydration (0.01%); unspecified (0.2%); and other means (0.06%).

### Mental disorders, substance misuse/addiction, physical health problems, and other contributing factors

Table 3 shows that compared to those age 65–74, the two older groups included lower percentages of individuals with a history of suicide attempt (s). About a third in all three age groups were reported to have shown depressed mood at the time of death, although the proportion was significantly higher in those age 75–84 than those age 65–74. However, two older groups had significantly lower percentages of individuals with mental disorders than in those age 65–74 (32.2% in those age 75–84 and 23.9% in those age 85+ vs. 41.6% in those age 65–74). Depressive disorder/dysthymia and anxiety disorders were most common in all three age groups. The two older groups also had smaller proportions of individuals with other substance misuse/addiction problems than the 65–74 age group. Overall, 51.2%, 64.5%, and 74.8% in the 65–74, 75–84, and 85+ age groups, respectively, did not have any reported depressed mood, mental disorders, or substance misuse/addiction problems. At the time of their injury, 22.7%, 16.0%, and 9.6% in the 65–74, 75–84, and 85+ age groups, respectively, were receiving mental health/substance use treatment.

**Table 3:** Mental disorders, substance misuse/addiction, and other contributing factors among suicide decedents age 65+: Results from single-variable multinomial logistic regression models.

	65–74 years *N* = 9,797 (54.7%)	75–84 years *N* = 5,592 (31.2%)	85+ years *N* = 2,528 (14.1%)	75–84 years vs. 65–74 years RRR (95% CI)	85+ years vs. 65–74 years RRR (95% CI)
History of suicide attempt (%)					
No, not available, unknown	87.4	92.1	94.5	–	–
Yes	12.6	7.9	5.5	0.60 (0.53–0.67)^***^	0.40 (0.33–0.48)^***^
Mental health and substance use problems					
Depressed mood at the time of injury (%)					
No, not available, unknown	68.3	66.5	68.1	–	–
Yes	31.7	33.5	31.9	1.08 (1.01–1.16)^*^	1.01 (0.92–1.11)
Current mental health disorder^1^ (%)					
No, not available, unknown	58.4	67.8	76.1	–	–
Yes	41.6	32.2	23.9	0.67 (0.62–0.72)^***^	0.44 (0.40–0.49)^***^
Types of disorder (among all decedents) (%)					
Depressive disorder/dysthymia	32.6	23.8	17.7		
Bipolar disorder	4.0	1.5	0.4		
Anxiety disorder	8.8	6.3	3.7		
Post-traumatic stress disorder	2.6	0.5	0.7		
Schizophrenia	1.3	0.6	0.1		
Dementia	0.3	0.6	0.7		
Other and unknown types of disorder	5.8	7.1	5.7		
Alcohol problem/addiction (%)					
No, not available, unknown	88.0	95.3	97.9	–	–
Yes	12.0	4.7	2.1	0.36 (0.31–0.41)^***^	0.16 (0.12–0.21)^***^
Other substance misuse^2^ (%)					
No, not available, unknown	95.0	98.5	99.4	–	–
Yes	5.0	1.5	0.6	0.30 (0.24–0.37)^***^	0.11 (0.06–0.18)^***^
Other addiction problem^3^ (%)					
No, not available, unknown	99.4	99.7	99.9	–	–
Yes	0.6	0.3	0.1	0.43 (0.24–0.77)^**^	0.20 (0.06–0.65)^**^
Summary of mental disorder^4^ and/or substance misuse/other addiction (%)					
No mental disorder or substance misuse/addiction	51.2	64.5	74.8	–	–
Substance misuse/addiction only	7.2	3.3	1.3	0.36 (0.31–0.43)^***^	0.12 (0.08–0.17)^***^
Mental disorder only	33.4	29.4	22.5	0.70 (0.65–0.75)^***^	0.46 (0.42–0.51)^***^
Both mental disorder and substance misuse /addiction	8.2	2.8	1.4	0.27 (0.22–0.32)^***^	0.12 (0.08–0.16)^***^
Mental health/substance misuse treatment receipt^5^ (%)					
At the time of injury	22.7	16.0	9.6	0.63 (0.58–0.68)^***^	0.36 (0.32–0.41)^***^
History of mental health treatment	28.5	20.0	12.5	0.65 (0.60–0.71)^***^	0.36 (0.34–0.42)^***^
Contributing physical health problem^6^ (%)					
No, not available, unknown	58.1	43.2	40.3	–	–
Yes	41.9	56.8	59.7	1.82 (1.70–1.95)^***^	2.05 (1.88–2.24)^***^
Contributing relationship and other life stressors					
Relationship problem^7^ (%)					
No, not available, unknown	82.7	88.8	93.4	–	–
Yes	17.3	11.2	6.6	0.60 (0.54–0.66)^***^	0.34 (0.28–0.40)^***^
Death/suicide of family/friend or traumatic anniversary (%)					
No, not available, unknown	91.1	88.1	86.3	–	–
Yes	8.9	11.9	13.7	1.37 (1.24–1.53)^***^	1.63 (1.42–1.86)^***^
Job-related or financial problem or eviction/loss of housing (%)					
No, not available, unknown	88.4	92.6	95.2	–	–
Yes	11.6	7.4	4.8	0.61 (0.54–0.69)^***^	0.39 (0.32–0.47)^***^
Criminal or civil legal problem (%)					
No, not available, unknown	95.3	97.9	99.3	–	–
Yes	4.7	2.1	0.7	0.43 (0.35–0.52)^***^	0.15 (0.09–0.23)^***^
Number of crises^8^, *M* (SE)	0.28 (0.01)	0.28 (0.01)	0.25 (0.01)	1.00 (0.94–1.06)	0.89 (0.82–0.97)^**^

1 Including disorders and syndromes listed in the Diagnostic and Statistical Manual of Mental Disorders, Fifth Edition (DSM-5) with the exception of alcohol and other substance dependence.

2 Including illicit drug use, except marijuana, even if addiction or abuse is not specifically mentioned. For marijuana, the use must be noted as chronic, abusive, or problematic.

3 An addiction other than alcohol or other substance that appears to have contributed to the death (e.g., gambling, sexual).

4 Including any mental health diagnosis.

5 Inclusive of pharmacotherapy, psychotherapy/counseling, any class (e.g., anger management) attendance, any facility-based care, and alcohol or narcotics anonymous.

6 Including any terminal/other illness, debilitating condition, chronic/acute pain, or other physical/functional issue (perceived, or diagnosed) that were relevant to suicide.

7 Problems with intimate partner and/or other family/relatives, other family stressors, caregiver burden, arguments, or abuse by a caregiver.

8 Total number of contributing factors that posed a crisis within 2 weeks of death.*p < 0.05; **p < 0.01; ***p < 0.001.

Those age 85 and older included the highest percentage (59.7%) of individuals with physical health problems as a contributing factor, followed by those age 75–84 (56.8%) and those age 65–74 (41.9%). The two older groups also included higher percentages of individuals with death/suicide of a family/friend or traumatic anniversary as a contributing factor, but lower percentages with other contributing factors. The average numbers of crises were small in all three age groups. Additional analysis showed that 76.7%, 75.2%, and 77.6% in the 65–74, 75–84, and 85+ age groups, respectively, had no crisis at the time of death.

### Correlates of intent disclosure in each age group: GLM results

Table 4 shows that depressed mood and physical health problems were significant correlates of intent disclosure in all three age groups: IRR = 1.44, 95% CI = 1.30–1.58 for those age 65–74, IRR = 1.42, 95% CI = 1.26–1.60 for those age 74–84, and IRR = 1.50, 95% CI = 1.26–1.79 for those age 85+ for depressed mood; and IRR = 1.42, 95% CI = 1.29–1.56 for those age 65–74, IRR = 1.61, 95% CI = 1.42–1.84 for those age 74–84, and IRR = 1.67, 95% CI = 1.38–2.04 for those age 85+ for physical health problems. Additionally, history of suicide attempt (s), mental disorder/substance misuse/addiction, relationship problems, death/suicide of family/friend, and the total number of crises were significant correlates in the 65–74 and 75–84 age groups.

**Table 4:** Correlates of intent disclosure (vs. no disclosure) in each age group: Results from generalized linear models with Poisson and log link.

	65–74 age group IRR (95% CI)	75–84 age group IRR (95% CI)	85+ age group IRR (95% CI)
History of suicide attempt	1.36 (1.20–1.54)^***^	1.47 (1.22–1.77)^***^	1.26 (0.91–1.74)
Depressed mood	1.44 (1.30–1.58)^***^	1.42 (1.26–1.60)^***^	1.50 (1.26–1.79)^***^
Mental disorder/substance misuse/other addiction: vs. no disorder/substance misuse			
Substance misuse/addiction only	1.36 (1.14–1.62)^**^	1.50 (1.14–1.97)^**^	1.05 (0.52–2.13)
Mental disorder only	1.31 (1.18–1.46)^***^	1.27 (1.11–1.44)^***^	1.19 (0.99–1.45)
Both mental disorder and substance misuse/addiction	1.35 (1.15–1.59)^***^	1.25 (0.91–1.72)	0.86 (0.40–1.83)
Physical health problem	1.42 (1.29–1.56)^***^	1.61 (1.42–1.84)^***^	1.67 (1.38–2.04)^***^
Relationship problem	1.28 (1.14–1.44)^***^	1.25 (1.05–1.49)^*^	1.32 (0.97–1.79)
Death/suicide of family/friend	1.22 (1.05–1.42)^*^	1.21 (1.01–1.44)^*^	1.19 (0.93–1.54)
Criminal or civil legal problem	1.13 (0.92–1.39)	1.23 (0.84–1.79)	1.68 (0.42–1.03)
Job-related, financial, or housing problem	1.04 (0.90–1.19)	0.94 (0.76–1.16)	0.66 (0.42–1.03)
No. of crises	1.13 (1.05–1.22)^**^	1.20 (1.09–1.33)^***^	1.05 (0.89–1.24)
Male	1.05 (0.93–1.19)	0.92 (0.77–1.10)	0.70 (0.53–0.93)^*^
Racial/ethnic group: vs. Non-Hispanic White			
Black/African American	0.84 (0.62–1.15)	0.82 (0.53–1.26)	1.41 (0.77–2.57)
Hispanic	0.92 (0.71–1.18)	0.74 (0.51–1.07)	0.88 (0.48–1.62)
Asian/Pacific Islander	0.61 (0.39–0.95)^*^	0.86 (0.56–1.32)	1.23 (0.76–1.99)
Other	0.97 (0.58–1.62)	0.74 (0.33–1.66)	0.74 (0.18–2.99)
Marital status: vs. Married			
Divorced/separated	1.01 (0.91–1.13)	1.03 (0.89–1.19)	1.02 (0.77–1.35)
Widowed	1.02 (0.87–1.19)	0.89 (0.76–1.04)	0.81 (0.67–0.99)^*^
Never married/single-unspecified	0.93 (0.79–1.09)	0.94 (0.70–1.25)	0.85 (0.52–1.39)
Unknown	0.81 (0.50–1.32)	0.98 (0.51–1.90)	0.59 (0.14–2.49)
Education: vs. =<High school			
Some college/associate’s degree	0.90 (0.81–1.02)	0.92 (0.78–1.07)	1.22 (0.96–1.54)
Bachelor’s degree or higher	0.97 (0.87–1.09)	0.85 (0.73–0.98)^*^	1.10 (0.91–1.35)
Unknown	1.02 (0.75–1.38)	1.04 (0.70–1.52)	0.91 (0.49–1.69)
Military service	1.02 (0.91–1.13)	0.92 (0.81–1.05)	0.97 (0.79–1.19)
	*N* = 9,797	*N* = 5,592	*N* = 2,528

* *p* < 0.05;

** *p* < 0.01;

*** *p* < 0.001.

In terms of demographic correlates, male sex and widowhood were associated with a lower likelihood of disclosure in the 85+ age group only; Asians/Pacific Islanders had a lower likelihood of disclosure in the 65–74 age group only; and those with a college or higher degree had a lower likelihood of disclosure in the 75–84 age group only.

### Associations between suicide means and intent disclosure and other characteristics

Table 5 shows that intent disclosure was not associated with firearm use and poisoning, but it was associated with a significantly lower likelihood of hanging/suffocation (IRR = 0.76, 95% CI = 0.68–0.86). Depressed mood was associated with a higher likelihood of hanging/suffocation and a lower likelihood of poisoning. Mental disorders, with or without substance misuse/addiction problems, and history of suicide attempt were associated with a lower likelihood of firearm use but a higher likelihood of hanging/suffocation and poisoning. Substance misuse/addiction problems were associated with a higher likelihood of poisoning.

**Table 5:** Association between intent disclosure and suicide means: Results from generalized linear models with Poisson and log link.

	Firearm vs. No firearm IRR (95% CI)	Hanging/suffocation vs. No hanging/suffocation IRR (95% CI)	Poisoning vs. No poisoning IRR (95% CI)
Disclosure of suicide intent	1.03 (0.98–1.08)	0.76 (0.68–0.86)^***^	1.06 (0.96–1.17)
History of suicide attempt	0.64 (0.59–0.70)^***^	1.43 (1.27–1.0)^***^	1.51 (1.36–1.67)^***^
Depressed mood	0.99 (0.95–1.03)	1.16 (1.06–1.27)^**^	0.88 (0.81–0.97)^**^
Mental disorder/substance misuse/other addiction: vs. no disorder/substance misuse			
Substance misuse/addiction only	0.96 (0.88–1.04)	0.91 (0.74–1.11)	1.46 (1.22–1.74)^***^
Mental disorder only	0.88 (0.85–0.92)^***^	1.36 (1.24–1.49)^***^	1.13 (1.03–1.25)^**^
Both mental disorder and substance misuse/addiction	0.83 (0.76–0.91)^***^	1.14 (0.95–1.37)	1.62 (1.40–1.88)^***^
Physical health problem	1.11 (1.07–1.16)^***^	0.67 (0.61–0.74)^***^	1.07 (0.98–1.17)
Relationship problem	1.09 (1.03–1.15)^**^	0.72 (0.63–0.83)^***^	1.12 (0.99–1.26)
Death/suicide of family/friend	1.00 (0.94–1.07)	0.94 (0.81–1.09)	1.14 (1.00–1.30)^*^
Criminal or civil legal problem	0.88 (0.79–0.98)^*^	1.62 (1.33–1.97)^***^	1.24 (0.98–1.56)
Job-related, financial, or housing problem	0.95 (0.89–1.02)	1.36 (1.19–1.54)^***^	0.95 (0.82–1.10)
No. of crises	1.02 (0.99–1.06)	0.91 (0.83–0.99)^*^	0.93 (0.85–1.01)
Age group: vs. 65–74 years			
75–84 years	1.06 (1.02–1.10)^**^	0.79 (0.71–0.87)^***^	0.86 (0.78–0.96)^**^
85+ years	1.00 (0.95–1.06)	0.96 (0.83–1.12)	0.95 (0.82–1.10)
Male	1.89 (1.77–2.02)^***^	1.09 (0.98–1.22)	0.24 (0.22–0.26)^***^
Racial/ethnic group: vs. Non-Hispanic White			
Black/African American	0.89 (0.79–1.00)	1.19 (0.92–1.54)	0.87 (0.67–1.14)
Hispanic	0.50 (0.43–0.57)^***^	3.42 (2.98–3.91)^***^	0.79 (0.61–1.03)
Asian/Pacific Islander	0.27 (0.21–0.35)^***^	4.31 (3.71–5.01)^***^	0.48 (0.34–0.68)^***^
Other	0.89 (0.72–1.10)	1.55 (1.03–2.35)^*^	1.05 (0.67–1.63)
Marital status: vs. Married			
Divorced/separated	0.93 (0.89–0.97)^**^	1.03 (0.93–1.15)	1.38 (1.25–1.53)^***^
Widowed	0.96 (0.91–1.01)	0.97 (0.85–1.10)	1.40 (1.25–1.57)^***^
Never married/single-unspecified	0.85 (0.79–0.91)^***^	1.27 (1.10–1.46)^**^	1.32 (1.13–1.54)^***^
Unknown	0.84 (0.69–1.02)	0.90 (0.60–1.34)	1.47 (0.98–2.22)
Education: vs. =<High school			
Some college/associate’s degree	0.98 (0.94–1.03)	1.03 (0.92–1.16)	1.07 (0.96–1.19)
Bachelor’s degree or higher	0.89 (0.85–0.93)^***^	1.09 (0.99–1.21)	1.37 (1.25–1.50)^***^
Unknown	0.98 (0.86–1.10)	1.07 (0.83–1.38)	0.92 (0.69–1.22)
Military service	1.11 (1.07–1.15)^***^	0.56 (0.50–0.63)^***^	0.80 (0.71–0.90)^***^
	*N* = 17,917	*N* = 17,917	*N* = 17,917

* *p* < 0.05;

** *p* < 0.01;

*** *p* < 0.001.

Physical health and relationship problems were associated with a higher likelihood of firearm use but lower likelihood of hanging/suffocation, whereas legal problems were associated with a lower likelihood of firearm use but a higher likelihood of hanging/suffocation. Death/suicide of family/friends was associated with a higher likelihood of poisoning only. Job-related, financial, or housing problems were associated with a higher likelihood of hanging/suffocation only, and more crises were associated with a lower likelihood of hanging/suffocation only.

The 75–84 age group was associated with a higher likelihood of firearm use but a lower likelihood of hanging/suffocation and poisoning. Male sex was associated with 1.89 (95% CI = 1.77, 2.02) likelihood of firearm use but 0.24 (95% CI = 0.22–0.26) likelihood of poisoning. Compared to non-Hispanic Whites, Hispanics and Asians/Pacific Islanders were less likely to have used firearms but 3–4 times more likely to have used hanging/suffocation. Asians/Pacific Islanders were also less likely to have used poisoning. Compared to married individuals, divorced/separated and never married individuals were less likely to have used firearms, but they and widowed individuals were more likely to have used poisoning. Never married individuals were also more likely to have used hanging/suffocation. Compared to those with high school or less education, those with a college or higher degree were less likely to have used firearms but more likely to have used poisoning.

## Discussion

We examined correlates of intent disclosure in three age groups of older suicide decedents and the associations between suicide means and intent disclosure and other characteristics. One out of five decedents 65 and older disclosed their suicide intent, but the 75–84 and 85+ age groups included slightly but statistically significantly higher proportions of disclosers than the 65–74 age group. Most disclosures were to family/friends, but one out of 10 disclosers age 85+ disclosed to a healthcare provider. As expected, an absolute majority of men, especially in the 75–84 and 85+ age groups, used firearms, but a higher proportion of women used poisoning than firearms.

Multivariable analysis results show that along with physical health problems, mental health/substance misuse/addiction problems, relationship problems, and death/suicide of family friends, and the number of crises were significant correlates of intent disclosure in the 65–74 and 75–84 age groups. On the other hand, for those age 85 years and older, only physical health problems were associated with a greater likelihood of intent disclosure. The findings partially support the study hypothesis. In the 65–74 and 75–84 age groups, sex was not a significant factor for intent disclosure; however, in the 85+ age group, men were significantly less likely to have disclosed their suicide intent. Significance of race/ethnicity and level of education also varied by age group. Multivariable analysis results show that intent disclosure was not associated with firearm use or poisoning as suicide methods but associated with lower odds of hanging/suffocation. Physical health problems, age 75–84 (compared to age 65–74), and male sex were associated with higher odds of firearm use, and mental health and/or substance misuse problems were associated with higher odds of hanging/suffocation and poisoning.

The overall disclosure rate in this study is lower than the disclosure rate found in the 2005–2014 NVDRS, as about a quarter of decedents age 70+ were found to have disclosed their intent during those earlier years (Choi et al., 2017). It is not clear if the lower rate in the present study reflects an actual decrease in disclosure in recent years or stems from some other factors related to NVDRS. We speculate that the lower disclosure rate in the present study may be due in part to the fact that most states in the Northeastern region began to participate in NVDRS in 2015 and, as described earlier, the disclosure rate was significantly lower in the region. Research is need to examine the reasons for regional variations in disclosure rates. Given that the number of NVDRS-participating states more than doubled between 2014 and 2017, the present study’s disclosure rate is likely to be more representative of the actual United States rate.

More importantly, the significance of physical health problems as a correlate of intent disclosure in all three age groups of older adults shows that intent disclosure (to mostly their family members) was a way of communicating their wish to end further physical suffering. Those who disclosed their intent to their healthcare providers may have done so to seek any medical advice; however, the data set does not contain details about the circumstances of the disclosure. A previous NVDRS-based study found that bodily pain and cancer were the most frequently mentioned physical health-related problems among older suicide decedents and that these older adults had often expressed their feelings of hopelessness and perceived burdensomeness, and longing for rest (Choi et al., 2019). Earlier studies of middle-aged and older individuals who attempted suicide also found a desire to escape from an unbearable situation, perceived burdensomeness, depression, experiences of defeat/powerlessness (especially among men), and lack of meaning in life were reasons/motivations for attempted suicide (Van Orden et al., 2015; Alessi et al., 2019). Unremitting pain and terminal illnesses can understandably trigger a sense of powerlessness and escape/rest motives. Under the circumstance, older adults, those age 85 years and older in particular, may feel that ending life on their own terms now rather than later (which is seen as inevitable) is justified and disclosing their suicide intent is one last willful act where they can exert control. A previous study of beliefs about late-life suicide showed that both older and younger adults rated suicide precipitated by physical health problems most positively as a rational, acceptable, and courageous act (Winterrowd et al., 2017). However, men age 85+ also had a lower likelihood of intent disclosure, suggesting that they tend to lack willingness to self-disclose.

The significant associations of intent disclosure with mental health/substance misuse problems, relationship problems, death/suicide of family/friend, and number of crises, in addition to physical health problems, in the 65–74 and 75+ age group also suggest a help-seeking motive among these older adults who were under multiple stressors. According to Alessi et al. (2019), interpersonal conflict and loss can engender anger, perceived loss of control, sense of defeat and abandonment, and grief, which can be aggravated by poor psychological resources and coping among those with mental health/substance misuse problems. Some older adults may welcome opportunities to receive support for reexamining their decision to die by suicide. The relatively small numbers of crises, on average, in all three age groups in the present study also show that most contributing factors were likely to have been an ongoing problem rather than a crisis.

The lack of significant association between intent disclosure and firearm and poisoning use is likely due to the fact that sex, regardless of the intent disclosure status, was the most significant determinant of these two methods of suicide. Sex was not a significant factor for hanging/suffocation, although race/ethnicity was. Hispanics and Asian/Pacific Islanders were significantly less likely than Non-Hispanic White to use firearms but they were 3–4 times more likely to use hanging/suffocation. These may be a reflection of the overall lower gun ownership rates among Hispanics and Asian/Pacific Islanders compared to non-Hispanic Whites (Pew Research Center, 2021). More research is needed to better understand the negative association between intent disclosure and hanging/suffocation.

As opposed to silent suicidal behaviors, intent disclosure provides a significant opportunity to prevent suicide. Although only one out of five older suicide decedents disclosed suicidal intent, this is not an insignificant proportion as timely interventions may have been able to prevent suicide. The higher likelihood of intent disclosure among all three age groups of older suicide decedents with physical health problems and mental health/substance misuse/addiction in the 65–74 and 75+ age groups have the following implications for suicide prevention. First, patient-centered/directed comfort and palliative care, including better pain management, is needed for older adults who perceive physical health problems as immutable and suicide as the only way to spare them further suffering. Special attention should be paid to older men suffering from physical health problems who may be at risk of suicide. As most of them may not disclose their suicidal intent, it may be necessary to thoroughly screen for and intervene against suicidal behavior. Healthcare services that recognize and support informal caregivers of older adults are also needed to equip those caregivers with practical tools and skill sets for detecting suicidal ideation/intent in their loved ones and implementing suicide prevention steps. Programs like Applied Suicide Intervention Skills Training (ASIST) have been found to save lives (Ashwood et al., 2015).

Second, older adults with mental health/substance misuse/addiction problems should be helped to access appropriate mental health and/or substance use treatment. A recent systematic review showed that suicide decedents who were men, under 25 years or 65 years and older, racial/ethnic minorities, residents in rural areas, and experienced stressors and used violent means of suicide were less likely to have received mental health services (Tang et al., 2022). Untreated depression in late life, especially among older adults with physical health problems and disability, has been associated with increased and persisting depressive symptom trajectories, further deterioration of physical function and cognitive health, and increased mortality (Andreescu et al., 2008; Kaup et al., 2016; Mirza et al., 2016; Agustini et al., 2022). Given the inconclusive evidence of effectiveness of antidepressants for late-life suicide prevention, mental health treatment should include physical activity and collaborative management for reduction of suicidal behaviors at integrated physical and behavioral health settings (Laflamme et al., 2022).

At-risk and problem alcohol use and/or other substance misuse/addiction can also aggravate depression and cognitive decline in late life (Blow et al., 2007; Topiwala and Ebmeier, 2018). Healthcare providers should screen for substance use and make treatment referrals. A study found that individuals age 65–69 were more likely to complete treatment successfully than those 75 or older; however, with age appropriate treatment, the latter group is also likely to have positive treatment experience (Sahker et al., 2015).

Third, for those who are despondent over relationship/other life stressors, mental health treatment should involve strengthening their coping skills and promoting positive psychological factors. Research has shown that interventions that emphasize reasons for living and meaning in life have positive impact on enhancing psychological well-being and protecting against suicidal behavior (Heisel et al., 2016).

Finally, older adults who disclosed suicidal intent or ideation should be restricted from accessing suicide means. Given the possibility of substitution of means (Florentine and Crane, 2010), both informal and formal caregivers should talk with the older adults and ideally, find mutually agreed-upon ways that can limit the older adults’ access to potential suicide means. Concurrently, older adults should also receive appropriate treatment and services for the conditions that triggered suicidal intent and thoughts. Formal and informal caregivers should pay special attention to men in their 80s suffering from physical health problems as they are less likely to self-disclose suicidal intent and significantly more likely to use lethal means, compared to older women.

This study had limitations. First, NVDRS data on intent disclosure was collected from the memory of older adults’ family and other informants, and thus, may have been subject to recall bias. Also, it was not possible to distinguish between those who did not disclose and those whose information on disclosure was not available. Second, NVDRS data on physical and mental health and substance misuse were also collected from decedents’ family/friends and other informants and/or suicide notes, not from healthcare providers. Without diagnosis data and input from older adults’ healthcare providers, the validity of these data may also be questionable. NVDRS allowed that contributing health problems may have been perceived and psychosomatic (influenced by depression and/or other affective or cognitive disorders) rather than formally diagnosed. Third, although a majority of states participated in the 2017–2019 NVDRS, some states did not provide data on all 3 years and others provided only partial data limited to some counties. Thus, the findings are not representative of all United States older-adult suicide decedents. Fourth, any theory testing had to be precluded given the lack of quantitative measures of any psychological state measures including hopelessness, thwarted belongingness, and perceived burdensomeness. NVDRS also does not include variables on whether or not any suicide prevention steps had been applied, especially by healthcare providers, following the intent disclosure prior to the fatal injury and the nature of the prevention, if any. The information would have been helpful to better understand the circumstances of the older adults’ suicide and provide further insights into potentially effective prevention strategies.

## Conclusion

This study found that one fifth of suicide decedents 65 years and older disclosed their suicide intent within a month of fatal injury. Both physical and mental health/substance misuse/addiction problems, relationship/other life stressors were associated with higher likelihood of intent disclosure among those under 85, but only physical health problems were associated with higher likelihood of intent disclosure among those 85 and older. Although sex was not a significant correlate of intent disclosure in those under 85, men 85 and older had lower likelihood of suicide intent disclosure, indicating the need for thorough screening and prevention strategies for older men with physical health problems who may be at risk of suicidal behavior. Intent disclosure was not associated with use of firearms and poisoning as suicide means but associated with lower likelihood of hanging/suffocation. Late-life suicide prevention strategies should include more patient-centered comfort and palliative care, mental health/substance misuse/addiction treatment, and restriction of access to potential suicide means for those who disclosed suicidal intent. More research is needed on older adults who disclose suicide intent and late-life suicide prevention strategies.

## Data availability statement

The data analyzed in this study is subject to the following licenses/restrictions: The authors received permission to use the United States CDC-collected data set based on a data use agreement. The data set is not publicly available. Requests to access these datasets should be directed to nvdrs-rad@cdc.gov.

## Ethics statement

This study based on de-identified/deceased individuals was exempt from the authors’ Institutional Review Board’s review. Ethical review and approval was not required for this study in accordance with the local legislation and institutional requirements. Written informed consent was not required for this study in accordance with the national legislation and the institutional requirements.

## Author contributions

Both authors listed have made a substantial, direct, and intellectual contribution to the work and approved it for publication.

## Funding

The authors received support from the University of Texas at Austin’s internal research fund.

## Conflict of interest

The authors declare that the research was conducted in the absence of any commercial or financial relationships that could be construed as a potential conflict of interest.

## Publisher’s note

All claims expressed in this article are solely those of the authors and do not necessarily represent those of their affiliated organizations, or those of the publisher, the editors and the reviewers. Any product that may be evaluated in this article, or claim that may be made by its manufacturer, is not guaranteed or endorsed by the publisher.
